# A Rare Case of Asymptomatic Solid Pseudopapillary Tumor of the Pancreas in a Pregnant Woman: A Conservative Management Approach

**DOI:** 10.7759/cureus.85454

**Published:** 2025-06-06

**Authors:** Abderrahman Atmani, Tijani El Harroudi, Tariq Bouhout, Badr Serji

**Affiliations:** 1 Department of Surgical Oncology, Mohammed VI University Hospital, Mohammed First University, Oujda, MAR; 2 Department of Surgical Oncology, Mohammed VI University Hospital, Regional Oncology Center, Oujda, MAR; 3 Department of Surgical Oncology, Faculty of Medicine and Pharmacy, Mohammed First University, Oujda, MAR

**Keywords:** asymptomatic, conservative treatment, pregnancy, solid pseudopapillary tumors, surgical case reports, young women

## Abstract

Solid pseudopapillary tumors (SPT) of the pancreas are considered rare pancreatic neoplasms with low-grade malignant potential and a favorable outcome after surgical resection. They are mainly observed in young women in their second and third decades of life. The association between SPT and pregnancy is exceptional. To the best of our knowledge, only a few cases of SPT discovered and resected during pregnancy have been reported in the English literature. In this case report, we discuss the clinical findings of a 19-year-old Moroccan woman who presented with an asymptomatic SPT during the 12th week of pregnancy and share our experience managing this situation during pregnancy.

## Introduction

Solid pseudopapillary tumor (SPT) of the pancreas is a low malignant potential neoplasm first described by Frantz in 1959 [1]. It represents approximately 1-2% of exocrine pancreatic tumors [2,3]. It predominantly affects young females, most commonly in the second or third decade of life, and often presents asymptomatically or with mild abdominal pain [4,5]. The World Health Organization (WHO) standardized its nomenclature in 1996 [4], recognizing its distinct pseudopapillary histology. Surgical resection remains a curative option, even in cases of metastatic disease [6]. SPT during pregnancy is exceptionally rare, with only four cases reported in English literature [7-10]. We present the fifth case, a 19-year-old primigravida with an 11 cm pancreatic head SPT, which was successfully managed via enucleation at 13 weeks of gestation. This case represents both the largest pregnancy-associated SPT successfully managed with conservative resection and the third reported instance of surgical intervention during gestation.

## Case presentation

**Figure 1:**
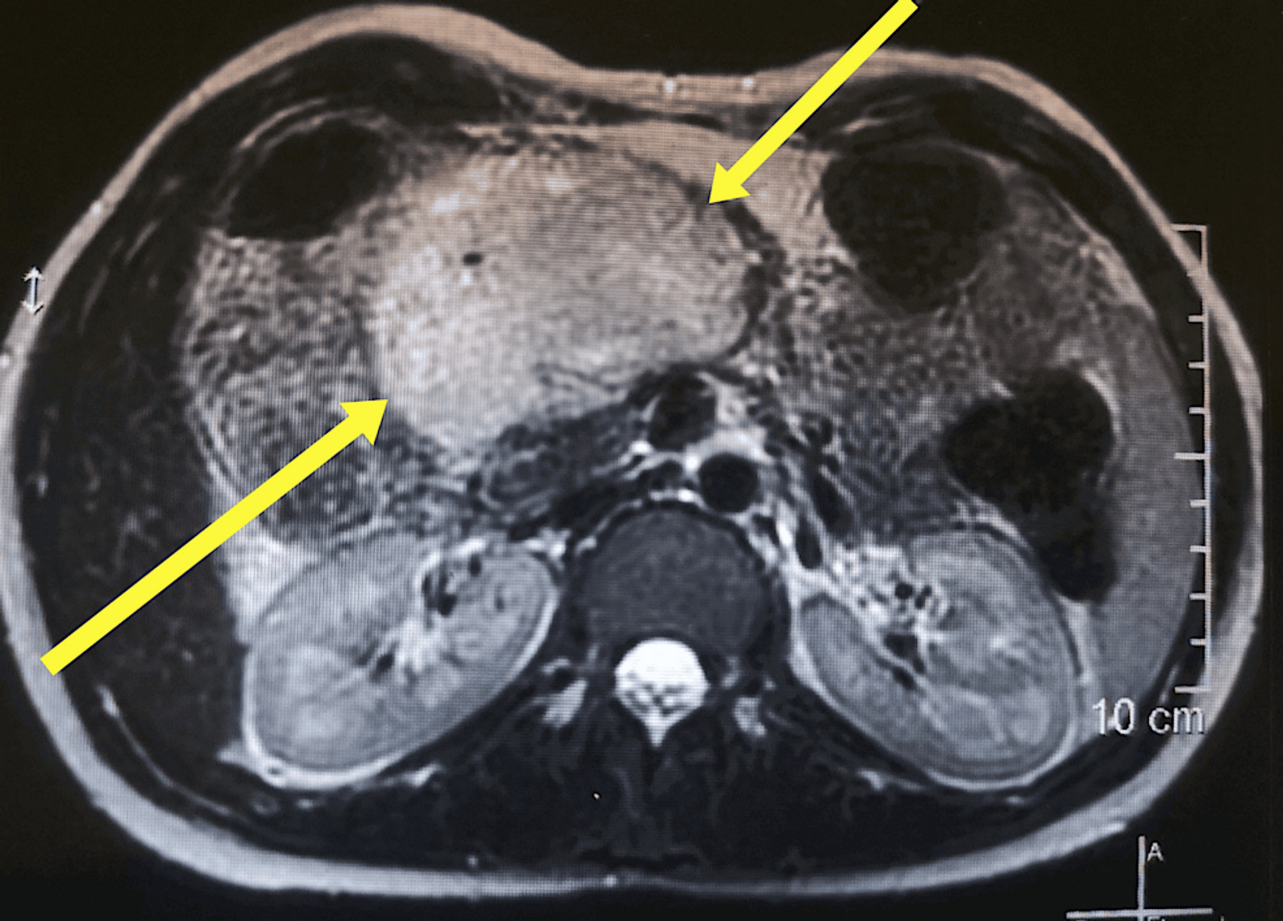
MRI aspect of the large solid-cystic mass of the head and pancreatic isthmus (yellow arrows)

A 19-year-old North African primiparous woman in the 12th week of pregnancy was referred by her gynecologist to our surgical oncology department, whose ultrasonography (US) for her regular prenatal care revealed, fortuitously, the presence of an abdominal mass. The patient has no particular personal pathological or family history. The physical examination of our patient found a healthy young woman, without weight loss, anicteric, with a soft mass in the epigastric/middle abdominal quadrant. The laboratory finding was normal. Due to pregnancy, the computed tomography (CT) was not performed. Therefore, magnetic resonance imaging (MRI) was performed and revealed the presence of a giant solid-cystic mass (110×110×70 mm) with a mixture of high and low signal intensity on T1- and T2-weighted images in the head and the isthmus of the pancreas, which was well limited and with a fibrous capsule (Figure 1).

The Wirsung duct and the common bile duct were preserved, with a mass effect on the splenic vein, spleno-mesenteric trunk, and superior mesenteric vein, which remained patent.

**Figure 2:**
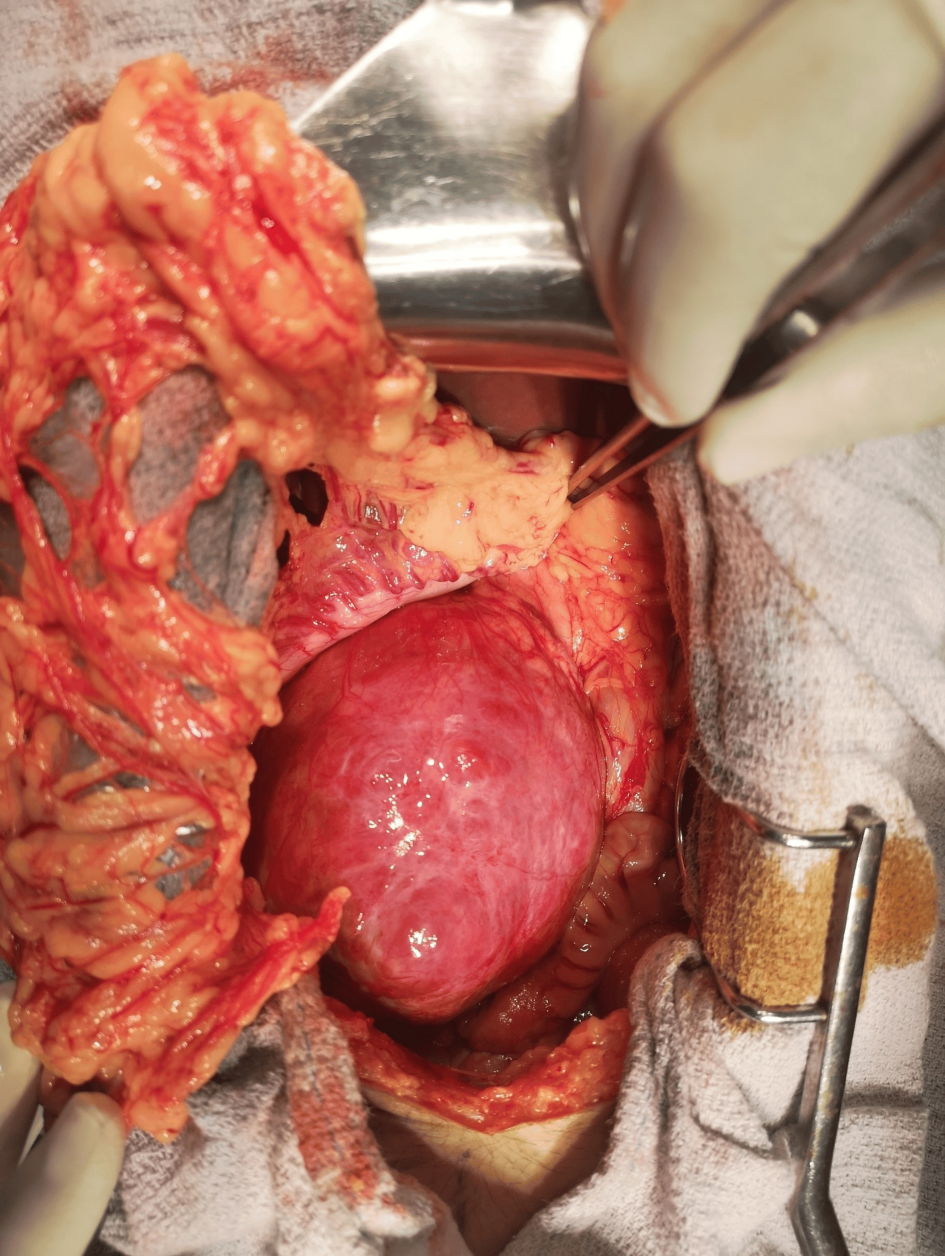
The intraoperative aspect of the lesion

**Figure 3:**
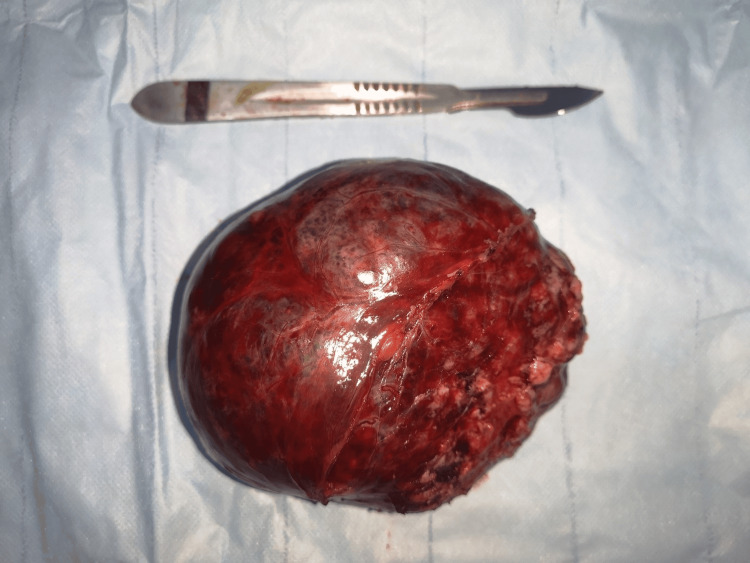
The postoperative aspect of the resected specimen

According to the imaging, the diagnosis of SPT was highly suspected. A multidisciplinary consultation was made, and the surgery was judged relatively safe. General anesthesia was proposed as a safe procedure in the perioperative period; prophylactic tocolysis was carried out by phloroglucinol/trimethylphloroglucinol 80 mg as a vaginal suppository three times on the day of the intervention, followed by 400 mg progesterone vaginal suppositories once a day for one month after the intervention. The surgery was performed in the 13th week of pregnancy. A median laparotomy was indicated and performed; there were no signs of metastatic lesions in the exploration, and due to the dense fibrous capsule, a conservative treatment was chosen, and a successful tumor enucleation was performed without any visible neoplastic tissue remaining at the operation site. The procedure was completed in three hours and 36 minutes of skin-to-skin operative time with an estimated blood loss of 70 mL (Figures 2, 3).

The patient was transferred back to the general surgery service, where she was successfully advanced to a soft diet. She was discharged home on postoperative day 11 with no complications. The pregnancy reached full term and resulted in an uncomplicated spontaneous vaginal delivery without anomalies. At the six-month postoperative follow-up, both mother and child remained healthy with no evidence of complications or tumor recurrence.

**Figure 4:**
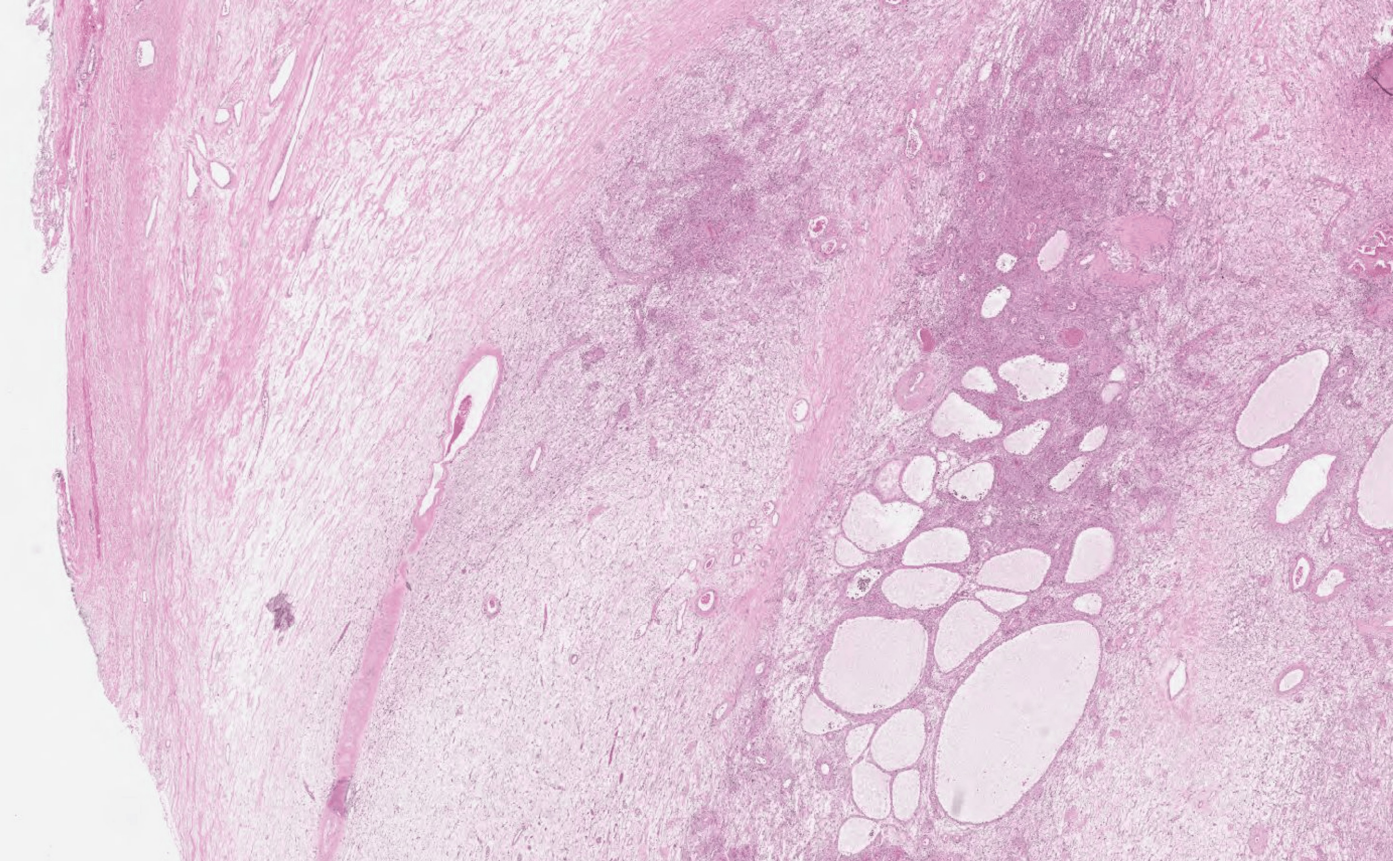
Microphotography showing a well-circumscribed proliferation made of hypo- and hypercellular zones. A microcystic architecture is focally observed (HE, 40×) HE: Hematoxylin and eosin stain

**Figure 5:**
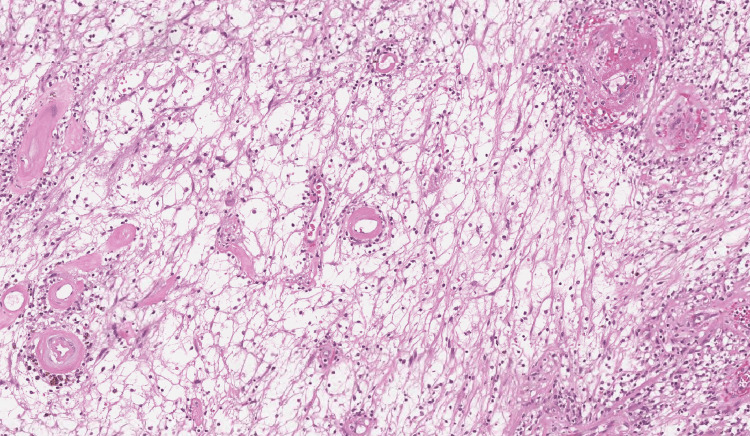
Microphotography showing a spindle cell proliferation made of bland Schwann cells. The vessels are dilated, made of a thick hyaline wall (HE, 200×) HE: Hematoxylin and eosin stain

**Figure 6:**
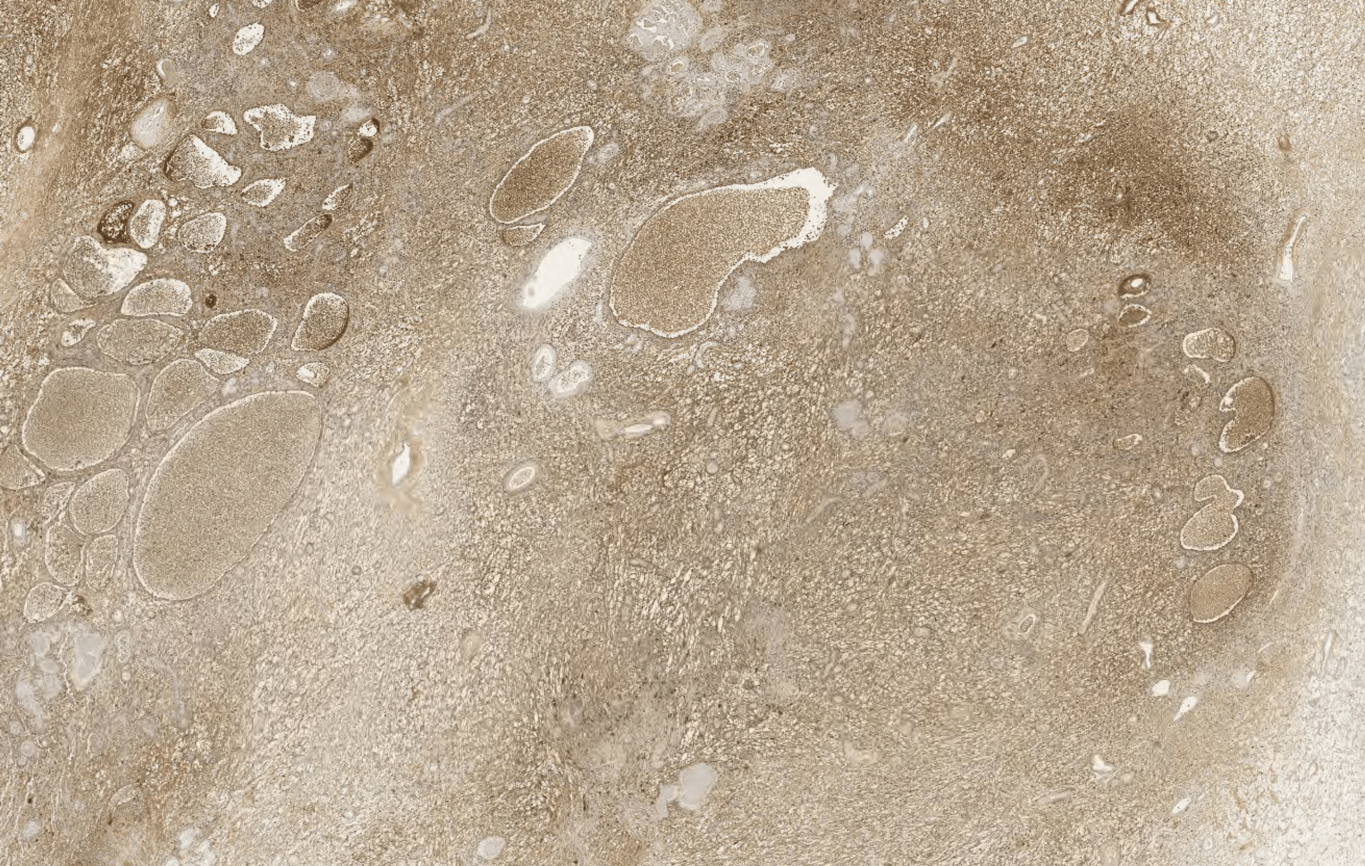
Tumor cells are stained with S-100 antibody immunohistochemistry

Histopathological examination showed tumoral proliferation disposed in solid masses and ramified pseudopapillary structures with central capillary and external double layers of cells (Figures 4-6). The immunohistochemistry results revealed that the tumor cells expressed vimentin and β-catenin, but not cytokeratin 7 (CK7) or chromogranin; therefore, the diagnosis of SPT was confirmed, which had been completely excised with clear margins.

## Discussion

SPT is a rare pancreatic neoplasm, representing 0.13-2.7% of all pancreatic masses [11]. The pathogenesis of SPT is yet to be determined, though accumulating evidence suggests a neuroendocrine origin [12-14]. Tumor development is typically slow and may be influenced by high levels of progesterone, particularly during pregnancy [5,15,16]. Our case of SPT in pregnancy is the fifth ever reported and the third to undergo surgical resection during pregnancy [7-10].

SPT presents with nonspecific symptoms. It is a non-functional, slow-progressing tumor that can achieve a considerable size before primary symptoms appear [17]. In a review of 718 SPT cases in English literature [5], a sex ratio of 9.78:1 and a mean age of 21.97 years were noted. Upper abdominal pain was the most frequent manifestation, occurring in 46.5% of cases, followed by a slowly growing mass in 34.8% of cases. Asymptomatic cases represented 15.5% of cases. The tumor was most commonly located in the tail (35.9%) and the head (34%) of the pancreas, followed by the body [5]. Our patient had the tumor in the head of the pancreas, consistent with previous reports [7,8]. Similar to the case reported by Al-Humairi et al., Salazar-Mejía et al. reported a case affecting the tail and body of the pancreas [9,10].

The size of the tumor at diagnosis in our case of SPT during pregnancy is the largest among those located in the head and treated by conservative surgery, compared to previously reported cases [7-10]. Notably, all the previously cited cases and 83% of cases reported by Papavramidis et al. had tumors larger than 5 cm [5]. Tumors larger than 100 mm in diameter were observed in 34% of cases in Papavramidis et al.'s series [5].

Preoperative diagnosis of SPT is challenging due to similarities with other pancreatic cystic lesions [5]. The first imaging technique is usually ultrasound, which shows a well-defined heterogeneous epigastric mass. This is followed by a CT scan as the next recommended imaging procedure. In our case, a CT scan was not performed due to the pregnancy, so an MRI was preferred. MRI characterized a large solid-cystic mass with T1- and T2-weighted images. The presence of SPT can be suspected if MRI reveals an encapsulated heterogeneous solid-cystic mass without an evident internal septum [18]. Neuroendocrine tumors and mucinous cystadenocarcinomas represent the main differential diagnoses for suspected SPT, particularly given the less favorable prognosis of the latter [5,19].

Histopathologically, SPT cells exhibit a notably uniform growth pattern, with pseudopapillary, solid, and/or hemorrhagic pseudocystic structures in various proportions [20]. SPTs and neuroendocrine tumors have similar histopathological patterns and overlapping immunohistochemical profiles [19]. β-catenin nuclear labeling in SPTs and E-cadherin staining in neuroendocrine tumors are the most specific and sensitive markers for these conditions. Chromogranin expression may be associated with SPTs rather than neuroendocrine tumors [21].

The treatment of SPT is primarily based on radical resection, even in cases with locally advanced extension or metastasis [5,22,23]. Surgical resection is usually feasible with both radical and conservative procedures, such as R0 enucleation, which are acceptable therapeutic strategies for SPT [24,25].

## Conclusions

Despite SPT being a rare neoplasm, the presence of a relatively large, well-limited, hemorrhagic, cystic pancreatic mass in a young female patient is sufficient criteria to suspect SPT. Our case provides additional evidence that SPT can occur during pregnancy and demonstrates the feasibility of performing a conservative surgical resection while preserving the life of the fetus. The multidisciplinary management of this entity between gynecologists, anesthesiologists, and surgeons, the right timing of the intervention, and the suitable surgical technique, followed by vigilant postoperative care, are all crucial factors for the success of the therapeutic approach.
